# Metabolic Benefits of Saroglitazar 4 mg in Type 2 Diabetes Mellitus With Dyslipidemia: A Retrospective Real-World Observational Cohort Study From India

**DOI:** 10.7759/cureus.111328

**Published:** 2026-06-22

**Authors:** Aravinda Jagadeesha, Supratik Bhattacharyya, Abhishek Shrivastava, Shambo S Samajdar, Gagan Gunjan, Shweta Bhardwaj, Shaishavi Telang

**Affiliations:** 1 Internal Medicine, Dr Aravind's Diabetes Centre, Bangalore, IND; 2 Endocrinology, Diabetes and Metabolism, SKN Diabetes and Endocrine Centre, Naihati, IND; 3 Medicine, RR Hormone Clinic, Jabalpur, IND; 4 Clinical and Experimental Pharmacology, Diabetes and Allergy-Asthma Therapeutics Specialty Clinic, Kolkata, IND; 5 General Medicine, Rajendra Institute of Medical Sciences, Ranchi, IND; 6 Medicine, Military Hospital, Agra, IND; 7 Medical Affairs, Zydus Helathcare Limited, Mumbai, IND

**Keywords:** diabetic dyslipidemia, hypertriglyceridemia, non-alcoholic fatty liver disease, pparα/γ agonist, real-world evidence, saroglitazar, type 2 diabetes mellitus

## Abstract

Background

This retrospective real-world study evaluated saroglitazar 4 mg in Indian adults with type 2 diabetes mellitus (T2DM) and dyslipidemia.

Methods

Six hundred patients receiving saroglitazar 4 mg at Dr Aravind’s Diabetes Centre, Bengaluru, were assessed at baseline, four months, and six months. Glycemic, lipid, hepatic, FibroScan, renal, and anthropometric parameters were analyzed using paired t-tests.

Results

Significant improvements were observed across all outcomes (p<0.0001). Fasting blood sugar (FBS) decreased by 101.81 mg/dL, postprandial blood sugar (PPBS) by 111.58 mg/dL, and HbA1c from 8.90% to 7.61%. Triglycerides reduced by 163.34 mg/dL, high-density lipoprotein cholesterol (HDL-C) increased by 9.25 mg/dL, and total cholesterol (TC) and low-density lipoprotein cholesterol (LDL-C) decreased. Alanine aminotransferase (ALT), aspartate aminotransferase (AST), controlled attenuation parameter (CAP) score, liver stiffness measurement (LSM), serum creatinine, estimated glomerular filtration rate (eGFR), and body weight also improved.

Conclusion

Saroglitazar 4 mg significantly improved glycemic, lipid, hepatic, renal, and weight-related outcomes over six months in patients with T2DM and dyslipidemia.

## Introduction

Type 2 diabetes mellitus (T2DM) is a major public health challenge in the twenty-first century, affecting about 537 million adults worldwide and being especially common in low- and middle-income countries (LMIC), particularly India [1]. India presently harbors 77 million people with T2DM and is projected to exceed 134 million by 2045, thereby positioning India as the second most affected nation globally following China [2]. The pathophysiology of T2DM is intricate and fundamentally associated with a broad array of metabolic abnormalities, including atherogenic dyslipidemia, hepatic steatosis, central obesity, and hypertension. This syndromic overlap significantly increases cardiovascular morbidity and mortality in affected individuals [3].

Diabetic dyslipidemia, a condition affecting 60-80% of individuals with T2DM [4-6], is a notable independent cardiovascular risk factor. This condition is characterized by elevated triglycerides, reduced high-density lipoprotein cholesterol (HDL-C), and the presence of small dense low-density lipoprotein (LDL) particles [4-6]. The underlying mechanism of dyslipidemia arises from insulin resistance-mediated dysregulation of hepatic lipid metabolism. This metabolic disruption results in increased secretion of very low-density lipoprotein (VLDL), impaired lipoprotein lipase activity, and, consequently, hypertriglyceridemia [7]. Conventional lipid-lowering therapies, particularly statins, demonstrate only modest efficacy in the triglyceride-HDL-C axis, which creates a significant therapeutic gap in this population.

Contemporary therapeutic options for triglyceride-rich dyslipidemia include omega-3 fatty acid formulations, fibrates, glucagon-like peptide-1 (GLP-1) receptor agonists, and newer lipid-modifying therapies such as Proprotein Convertase Subtilisin/Kexin Type 9 (PCSK9) inhibitors [8,9]. Omega-3 fatty acid formulations and fibrates primarily reduce triglyceride levels, while GLP-1 receptor agonists may improve body weight, glycemic control, and overall cardiometabolic risk, with potential favorable effects on triglyceride-rich lipoproteins [8]. PCSK9 inhibitors are primarily LDL-C-lowering agents, although modest effects on triglycerides and HDL-C have also been reported [9]. Despite these options, a therapeutic need remains for agents that can simultaneously address hypertriglyceridemia, glycemic dysregulation, and hepatic metabolic abnormalities in patients with T2DM and mixed dyslipidemia.

The peroxisome proliferator-activated receptors (PPARs), which are nuclear transcription factors, play a key role in regulating lipid metabolism, glucose homeostasis, and inflammatory pathways. PPARα activation reduces hepatic triglyceride synthesis and promotes fatty acid oxidation, whereas PPARγ activation enhances insulin sensitivity and peripheral glucose uptake [10-12]. In 2013, saroglitazar (Lipaglyn®, Zydus Lifesciences, India), the first member of the glitazar class, received regulatory approval in India as a novel PPARα/γ dual agonist with predominant PPARα and moderate PPARγ activity [13-15]. Its dual agonism offers the mechanistic basis for simultaneously targeting the glycemic and lipid abnormalities of T2DM without the adverse effects historically associated with pure PPARγ agonists, such as fluid retention and weight gain [16].

Evidence from existing clinical trials on saroglitazar, including the pivotal Prospective Randomized Efficacy and Safety Study (PRESS) V and PRESS VI studies, as well as several observational real-world studies, has demonstrated significant improvements in triglycerides, HbA1c, and hepatic parameters [14,15,17]. However, most available real-world studies have been limited by smaller sample sizes, shorter follow-up durations, single-domain outcome assessment, or restricted evaluation of hepatic and renal parameters [18-20]. Furthermore, comprehensive Indian real-world data integrating glycemic control, detailed lipid profiling, FibroScan® (Echosens, Paris, France) derived markers of hepatic steatosis and fibrosis, anthropometric measures, and renal outcomes in a large patient cohort remain limited [20].

Therefore, the present retrospective observational cohort study was undertaken to comprehensively evaluate the real-world metabolic effects of saroglitazar 4 mg over six months in 600 Indian patients with T2DM and dyslipidemia. The study integrates glycemic, lipid, hepatic, FibroScan-derived controlled attenuation parameter (CAP) score, and liver stiffness measurement (LSM), anthropometric, and renal outcomes, thereby providing a broad real-world assessment of saroglitazar in a high-burden clinical setting.

## Materials and methods

Study design and setting

This was a retrospective, single-arm, observational cohort study conducted at Dr Aravind’s Diabetes Centre, Bengaluru, India. Patients who had been prescribed saroglitazar (4 mg) at baseline and had documented follow-up data available for six months, from September 2024 to March 2025, were reviewed and included in the study.

Study participants

The following eligibility criteria were applied to identify medical records for inclusion in the study: (i) adults aged ≥ 18 years; (ii) confirmed T2DM diagnosis with dyslipidemia; fasting triglycerides ≥150 mg/dL and/or HDL-C below target (men <40 mg/dL; women <50 mg/dL); (iii) patients with comorbidities such as hypertension and obesity; (iv) patients whose medical records documented receipt of saroglitazar 4 mg/day, with available baseline and follow-up glycemic and lipid profile values, in addition to ongoing antidiabetic and/or statin therapy for ≥three months. The exclusion criteria were type 1 diabetes mellitus; severe hepatic failure, active hepatic disease [alanine aminotransferase (ALT) or aspartate aminotransferase (AST)] > 3× upper limit of normal at screening); severe renal impairment [estimated glomerular filtration rate (eGFR) <30 mL/min/1.73m²]; New York Heart Association (NYHA) class III or IV heart failure; malignancy; pregnancy or lactation; documented hypersensitivity to saroglitazar or any PPAR agonist; and patients lost to follow-up.

Body mass index (BMI) categories were defined as underweight <18.5 kg/m², normal weight 18.5-24.9 kg/m², overweight 25.0-29.9 kg/m², and obesity ≥30.0 kg/m². Obesity recorded as a comorbid condition was based on physician documentation in the clinical record and may have included central obesity or prior clinical diagnosis, whereas BMI-defined obesity was analyzed separately using the BMI ≥30.0 kg/m² threshold. Hypertension was defined as a documented physician diagnosis, use of antihypertensive medication, or recorded blood pressure ≥140/90 mmHg in the medical record.

Intervention

All patients had been prescribed saroglitazar 4 mg orally once daily in addition to routine antidiabetic therapy, including metformin, sulfonylureas, DPP-4 inhibitors, thiazolidinediones (TZDs), SGLT2 inhibitors, insulin, statins, and other disease-specific medications. These medications had been continued without dose modification unless clinically indicated. The dose of saroglitazar was recorded as unchanged throughout the study period.

Outcome measures

Demographic details (age, gender, height, body weight), clinical profile (duration of T2DM in years, existing treatment, comorbid conditions), and laboratory measurements (glycemic, lipid, hepatic, and renal parameters) were documented. The primary outcomes were changes in glycemic parameters such as fasting blood sugar (FBS) mg/dL, postprandial blood sugar (PPBS) mg/dL, glycated hemoglobin (HbA1c, %), and changes in the full lipid profile including total cholesterol (TC) mg/dL, LDL-C (mg/dL), HDL-C (mg/dL), and triglycerides (TG) mg/dL. Secondary outcomes included changes in hepatic parameters such as ALT (U/L), AST (U/L), CAP score (dB/m) measured by FibroScan® as a validated non-invasive marker of hepatic steatosis, and LSM (kPa) by vibration-controlled transient elastography (VCTE) as an index of hepatic fibrosis. FibroScan-derived CAP score and LSM were collected from routine clinical records where available. CAP was used as a non-invasive marker of hepatic steatosis, and LSM was used as a marker of liver stiffness or fibrosis risk. Because of the retrospective design, detailed FibroScan quality parameters, including operator experience, probe type, number of valid measurements, interquartile range (IQR)/median ratio, and technical success rate, were not consistently documented. Anthropometric parameters such as body weight (kg) and BMI (kg/m²), and renal indices such as serum creatinine (mg/dL) and estimated glomerular filtration rate [eGFR, mL/min/1.73 m², by the chronic kidney disease epidemiology collaboration (CKD-EPI) formula] were also assessed. Assessments were conducted at three time points, which included baseline (pre-treatment), Visit 1 (four months post-initiation), and Visit 2 (six months post-initiation).

Statistical analysis

All data were expressed as mean ± standard deviation (SD) for continuous variables and as frequency and percentage for categorical variables. Within-patient comparisons between baseline and follow-up visits were performed using paired t-tests to assess clinically relevant changes from baseline to Visit 1 and Visit 2. Given that three repeated measurements were available, repeated-measures analysis of variance (ANOVA) or mixed-effects modeling may provide a more comprehensive assessment of within-subject correlations across time. Therefore, the paired t-test results should be interpreted as exploratory within-patient comparisons. A two-tailed p-value of <0.05 was considered statistically significant. Statistical analyses were performed using SPSS version 25.0 (IBM Corporation, Armonk, NY, USA).

Ethical approval

The study was conducted in accordance with the Declaration of Helsinki and applicable regulatory requirements. Institutional ethics committee approval (CIEC/2025/04, dated 01 April 2025) was obtained before data collection. In addition, a waiver of informed consent was granted by the ethics committee, given the retrospective nature of the study and the use of de-identified medical record data.

## Results

Baseline characteristics

A total of six hundred patients were enrolled and assessed at all three time points. The mean age of the cohort was 51.9 ± 12.0 years, with a higher number of males than females (321 males, 53.5%; 279 females, 46.5%). The mean duration of T2DM was 6.2 ± 2.6 years. The mean baseline body weight was 70.71 ± 12.94 kg, and the mean BMI was 27.59 ± 5.17 kg/m². BMI category distribution revealed that 36.5% of patients were overweight (25.0-29.9 kg/m²), 29.7% were obese (≥30.0 kg/m²), 32.2% had normal weight, and 1.7% were underweight; collectively, 66.2% had a BMI ≥25 kg/m². Hypertension was present in 97.3% (n=584) and obesity in 86.0% (n=516) of patients, underscoring the high metabolic syndrome burden. It should be noted that BMI-defined obesity and obesity recorded as a comorbid condition represent different classifications. BMI-defined obesity, using BMI ≥30.0 kg/m², was present in 29.7% of participants, whereas obesity as a recorded comorbidity was present in 86.0% of participants and reflected physician documentation in the clinical records. The recorded comorbidity definition may have included central obesity or a prior clinical diagnosis of obesity. Concomitant antidiabetic therapy was comprehensive: all patients received metformin, sulfonylureas, and DPP-4 inhibitors; 98.3% received SGLT2 inhibitors; 26.2% required insulin; and 19.5% were on pioglitazone. Statin therapy was universal at baseline. Demographic and baseline characteristics are detailed in Table 1.

**Table 1:** Baseline demographic and clinical characteristics of the study cohort (n = 600) Data are presented as Mean ± SD for continuous variables and n (%) for categorical variables. SD: standard deviation; BMI: body mass index; T2DM: type 2 diabetes mellitus; DPP-4: dipeptidyl peptidase-4; SGLT2: sodium-glucose cotransporter-2.

Parameter	Mean ± SD	n (%)
Age (years)	51.9 ± 12.0	—
Height (cm)	160.4 ± 9.0	—
Weight (kg)	70.71 ± 12.94	—
BMI (kg/m²)	27.59 ± 5.17	—
Duration of T2DM (years)	6.2 ± 2.6	—
Gender Distribution		
Male	—	321 (53.5%)
Female	—	279 (46.5%)
BMI Category		
Underweight (<18.5 kg/m²)	—	10 (1.7%)
Normal (18.5–24.9 kg/m²)	—	193 (32.2%)
Overweight (25.0–29.9 kg/m²)	—	219 (36.5%)
Obese (≥30.0 kg/m²)	—	178 (29.7%)
Comorbid Conditions		
Hypertension	—	584 (97.3%)
Obesity	—	516 (86.0%)
Concomitant Antidiabetic Medications		
Metformin	—	600 (100%)
Sulfonylureas	—	600 (100%)
DPP-4 Inhibitors	—	600 (100%)
SGLT2 Inhibitors	—	590 (98.3%)
Thiazolidinediones (Pioglitazone)	—	117 (19.5%)
Insulin	—	157 (26.2%)
Statin Therapy	—	600 (100%)

Glycemic outcomes

Saroglitazar 4 mg produced progressive and statistically significant improvements in all three glycemic parameters across both follow-up visits (Table 2). FBS declined from a baseline of 268.84 ± 68.99 mg/dL to 222.08 ± 52.99 mg/dL at Visit 1 and further to 167.03 ± 46.18 mg/dL at Visit 2 - representing an absolute reduction of 101.81 mg/dL (−37.9%) from baseline to six months. PPBS decreased substantially with a reduction of 111.58 mg/dL (−41.2%), from 270.92 ± 103.78 mg/dL at baseline to 159.34 ± 54.51 mg/dL at Visit 2. Furthermore, HbA1c decreased from 8.90 ± 1.86% at baseline to 8.50 ± 1.81% at Visit 1 and 7.61 ± 1.55% at Visit 2, which corresponds to an absolute decrease of 1.30 percentage points (−14.6%). All changes were statistically significant (p<0.0001, paired t-test). The progressive decrease from Visit 1 to Visit 2 across all parameters confirms a sustained, time-dependent improvement in glycemic outcomes.

**Table 2:** Changes in glycemic parameters from baseline to Visit 1 (4 months) and Visit 2 (6 months) All p-values by paired t-test. Values expressed as Mean ± SD. B: baseline; FBS: fasting blood sugar; HbA1c: glycated hemoglobin; PPBS: postprandial blood sugar; SD: standard deviation; V2: Visit 2.

Parameter	Baseline (Mean ± SD)	Visit 1 4 months (Mean ± SD)	Visit 2 6 months (Mean ± SD)	Absolute Change (B→V2)	t-value (df = 599)	p-value
FBS (mg/dL)	268.84 ± 68.99	222.08 ± 52.99	167.03 ± 46.18	−101.81 (−37.9%)	64.91	<0.0001
PPBS (mg/dL)	270.92 ± 103.78	213.41 ± 76.34	159.34 ± 54.51	−111.58 (−41.2%)	42.56	<0.0001
HbA1c (%)	8.90 ± 1.86	8.50 ± 1.81	7.61 ± 1.55	−1.30 (−14.6%)	32.48	<0.0001

Lipid profile outcomes

Saroglitazar demonstrated a statistically significant improvement across all four lipid parameters after six months, as shown in Table 3. The most clinically relevant effect was observed in triglycerides, which decreased by 163.34 mg/dL (−48.9%) from a markedly elevated baseline of 334.04 ± 59.55 mg/dL to 170.70 ± 28.17 mg/dL at Visit 2, thereby approaching normal levels. HDL-C showed a considerable increase from a critically low baseline of 30.81 ± 6.42 mg/dL to 40.05 ± 5.67 mg/dL at Visit 2, which represents an absolute increase of 9.25 mg/dL (+30.0%) - a clinically relevant improvement considering the cardiovascular protective threshold. Furthermore, total cholesterol was reduced by 44.23 mg/dL (−22.6%) from 195.80 ± 41.62 mg/dL to 151.57 ± 29.97 mg/dL. LDL-C showed a modest but statistically significant reduction of 6.95 mg/dL (−4.9%), from 143.21 ± 62.57 mg/dL to 136.25 ± 63.18 mg/dL. All lipid parameter changes achieved p<0.0001.

**Table 3:** Changes in lipid profile parameters from baseline to Visit 1 (4 months) and Visit 2 (6 months) Values expressed as Mean ± SD. All p-values by paired t-test. SD: standard deviation; B: baseline; V2: Visit 2; LDL-C: low-density lipoprotein cholesterol; HDL-C: high-density lipoprotein cholesterol.

Parameter	Baseline (Mean ± SD)	Visit 1 4 months (Mean ± SD)	Visit 2 6 months (Mean ± SD)	Absolute Change (B→V2)	t-value (df = 599)	p-value
Total Cholesterol (mg/dL)	195.80 ± 41.62	178.62 ± 40.08	151.57 ± 29.97	−44.23 (−22.6%)	47.98	<0.0001
LDL-C (mg/dL)	143.21 ± 62.57	139.87 ± 62.89	136.25 ± 63.18	−6.95 (−4.9%)	4.94	<0.0001
HDL-C (mg/dL)	30.81 ± 6.42	35.54 ± 6.07	40.05 ± 5.67	+9.25 (+30.0%)	66.86	<0.0001
Triglycerides (mg/dL)	334.04 ± 59.55	261.39 ± 49.82	170.70 ± 28.17	−163.34 (−48.9%)	103.72	<0.0001

Hepatic parameters

Table 4 shows significant improvements in both hepatic enzyme levels and non-invasive FibroScan-derived markers of steatosis and fibrosis. ALT, the primary biomarker of hepatocellular injury, decreased by 14.50 U/L (−47.1%), from a baseline of 30.78 ± 4.54 U/L to 16.28 ± 3.23 U/L at Visit 2. AST declined by 16.97 U/L (−26.6%), from 63.69 ± 13.69 U/L to 46.72 ± 9.50 U/L. The CAP score, a validated measure of hepatic steatosis, decreased by 13.48 dB/m (−8.5%) from a baseline of 159.06 ± 73.57 dB/m to 145.59 ± 68.83 dB/m at Visit 2. Additionally, LSM, which indicates hepatic fibrosis/stiffness, decreased by 0.92 kPa (−10.6%), from 8.66 ± 2.87 kPa to 7.74 ± 2.25 kPa. All hepatic outcome changes were statistically significant (p<0.0001).

**Table 4:** Changes in hepatic parameters (liver enzymes and FibroScan indices) from baseline to Visit 1 (4 months) and Visit 2 (6 months) Values expressed as Mean ± SD. All p-values by paired t-test. SD: standard deviation; B: baseline; V2: Visit 2; ALT: alanine aminotransferase; AST: aspartate aminotransferase; CAP: controlled attenuation parameter (FibroScan®, hepatic steatosis); LSM: liver stiffness measurement (vibration-controlled transient elastography, hepatic fibrosis). CAP score was analyzed as a continuous variable at baseline, Visit 1, and Visit 2. The proportions of patients with normal CAP scores and mild, moderate, or severe steatosis categories were not available in the extracted retrospective dataset.

Parameter	Baseline (Mean ± SD)	Visit 1 4 months (Mean ± SD)	Visit 2 6 months (Mean ± SD)	Absolute Change (B→V2)	t-value (df = 599)	p-value
ALT (U/L)	30.78 ± 4.54	23.66 ± 3.80	16.28 ± 3.23	−14.50 (−47.1%)	143.63	<0.0001
AST (U/L)	63.69 ± 13.69	55.50 ± 11.23	46.72 ± 9.50	−16.97 (−26.6%)	55.27	<0.0001
CAP Score (dB/m)	159.06 ± 73.57	152.71 ± 71.40	145.59 ± 68.83	−13.48 (−8.5%)	8.41	<0.0001
LSM (kPa)	8.66 ± 2.87	8.36 ± 2.90	7.74 ± 2.25	−0.92 (−10.6%)	14.79	<0.0001

Anthropometric and renal outcomes

Changes in anthropometric and renal parameters from baseline to Visit 1 (4 months) and Visit 2 (6 months) are shown in Table 5. Body weight demonstrated a transient, negligible increase of 0.30 kg at Visit 1, followed by a net reduction of 0.96 kg (−1.3%) at Visit 2 compared to baseline (from 70.71 ± 12.94 kg to 69.75 ± 12.62 kg; p<0.0001). A decrease in BMI from 27.59 ± 5.17 kg/m² to 27.23 ± 5.11 kg/m² (−0.36 kg/m², −1.3%) was also observed. Renal function demonstrated a clinically significant improvement: serum creatinine decreased by 0.16 mg/dL (−18.0%), from a baseline of 0.87 ± 0.40 mg/dL to 0.71 ± 0.16 mg/dL at Visit 2. In addition, eGFR increased by 8.86 mL/min/1.73 m² (+9.1%), from 97.53 ± 45.17 mL/min/1.73 m² to 106.39 ± 20.89 mL/min/1.73 m². Both changes were highly statistically significant (p<0.0001).

**Table 5:** Changes in anthropometric and renal parameters from baseline to Visit 1 (4 months) and Visit 2 (6 months) Values expressed as Mean ± SD. All p-values by paired t-test. SD: standard deviation; B: baseline; V2: Visit 2; BMI: body mass index; eGFR: estimated glomerular filtration rate [chronic kidney disease epidemiology collaboration (CKD-EPI) formula].

Parameter	Baseline (Mean ± SD)	Visit 1 4 months (Mean ± SD)	Visit 2 6 months (Mean ± SD)	Absolute Change (B→V2)	t-value (df = 599)	p-value
Body Weight (kg)	70.71 ± 12.94	71.01 ± 12.71	69.75 ± 12.62	−0.96 (−1.3%)	5.80	<0.0001
BMI (kg/m²)	27.59 ± 5.17	27.71 ± 5.13	27.23 ± 5.11	−0.36 (−1.3%)	5.42	<0.0001
Serum Creatinine (mg/dL)	0.87 ± 0.40	0.75 ± 0.30	0.71 ± 0.16	−0.16 (−18.0%)	14.14	<0.0001
eGFR (mL/min/1.73 m²)	97.53 ± 45.17	107.33 ± 38.32	106.39 ± 20.89	+8.86 (+9.1%)	7.35	<0.0001

## Discussion

This large-scale, retrospective real-world cohort study of saroglitazar 4 mg demonstrated robust, multi-domain metabolic improvements across glycemic, lipid, hepatic, renal, and anthropometric parameters over six months in 600 Indian patients with T2DM and dyslipidemia. The study consistently achieved statistical significance (p<0.0001) across all measured outcomes, thereby providing compelling real-world evidence supporting saroglitazar's place in the management of the complex metabolic phenotype that characterizes T2DM in India.

HbA1c reduction of 1.30 percentage points (−14.6%), FBS reduction of 37.9%, and PPBS reduction of 41.2% were of statistical and clinical relevance. The American Diabetes Association (ADA) standards of care define a clinically meaningful HbA1c reduction as ≥0.5%, whereas the 1.30% reduction reported in this study exceeds this threshold substantially [21,22]. Chatterjee et al., in an observational study, reported an HbA1c reduction of 1.13%, while the PRESS XII randomized study demonstrated a reduction of 1.47% over 56 weeks with saroglitazar 4 mg [19,23]. The magnitude of improvement observed in this study could be attributed to the higher baseline HbA1c (8.90%) in the cohort. This is consistent with the well-established regression-to-the-mean effect, whereby glucose-lowering agents typically achieve greater absolute reductions at higher baseline glycemic levels.

Triglycerides decreased from 334.04 to 170.70 mg/dL, with a 48.9% reduction, which is the most significant individual lipid change and aligns with the primary pharmacological action of PPARα agonism, which activates lipoprotein lipase, reduces VLDL synthesis, and increases fatty acid β-oxidation [24,25]. The triglyceride reduction in this study is comparable with the PRESS V trial, which showed triglyceride reductions of about 45% with saroglitazar 4 mg over 24 weeks [14]. In addition, the real-world analysis by Kaul et al. reported triglyceride reductions of ~45-62% across 18 studies [17]. The 30% increase in HDL-C (from 30.81 to 40.05 mg/dL) is particularly significant, given the critically low baseline level and the cardiovascular risk associated with HDL-C in T2DM [26]. The reduction in total cholesterol (−22.6%) and LDL-C (−4.9%), achieved while patients were on statin therapy, further underscores saroglitazar's lipid-modifying activity and its consistency with prior studies [14,17]. The management of triglyceride-rich dyslipidemia in T2DM increasingly includes several therapeutic options beyond statins. Omega-3 fatty acid formulations and fibrates can reduce triglyceride levels, while GLP-1 receptor agonists may improve weight, glycemic control, and overall cardiometabolic risk [8]. PCSK9 inhibitors are mainly used for LDL-C reduction, with additional modest effects on other lipid fractions [9]. In this context, saroglitazar remains mechanistically relevant because its predominant PPARα and moderate PPARγ activity may address hypertriglyceridemia, insulin resistance, and hepatic metabolic dysfunction together [10-15]. However, direct comparative conclusions cannot be drawn from the present single-arm study.

The hepatic findings from this study add to the growing real-world evidence on saroglitazar use in patients with T2DM and metabolic dysfunction-associated steatotic liver disease [27-31]. Improvements were observed in ALT, AST, CAP score, and LSM over six months, consistent with previous real-world and clinical evidence reporting favorable hepatic enzyme and FibroScan-related outcomes with saroglitazar [20,28-32]. However, these findings should be interpreted cautiously because the study was retrospective, single-arm, and conducted in a heavily treated population. In particular, 98.3% of patients were receiving SGLT2 inhibitors, which may also improve hepatic steatosis and metabolic outcomes [33-35]. Therefore, the observed hepatic improvements should be considered associative and hypothesis-generating rather than definitive evidence of an independent saroglitazar-specific effect.

The observed improvements in renal function, including reduction in serum creatinine and increase in eGFR, are encouraging and add to the overall favorable metabolic profile observed in this real-world cohort [33-35]. Although most patients were receiving SGLT2 inhibitors and other background standard-of-care therapies, these findings suggest that saroglitazar 4 mg was associated with beneficial renal trends when used as part of comprehensive cardiometabolic management.

Body weight reduction was modest (−1.3%, −0.96 kg) compared with the pronounced weight loss expected with SGLT2 inhibitors [33]. This is consistent with saroglitazar's neutral-to-modest weight-loss profile reported across real-world studies [17,18]. The absence of significant weight gain, despite the PPARγ component of saroglitazar's activity, distinguishes it favorably from earlier glitazones and supports its acceptable metabolic risk profile in a polypharmacy setting [14,16].

Pioglitazone was used by 117 patients (19.5%) in this cohort. Because pioglitazone has PPARγ-mediated metabolic effects, there is potential overlap with the moderate PPARγ activity of saroglitazar. Pioglitazone users were not excluded because this study aimed to reflect routine, real-world clinical practice. However, formal subgroup analysis according to pioglitazone use was not performed, and this should be considered when interpreting the results.

The retrospective design, large sample size (n=600), comprehensive multiparameter assessment including validated FibroScan indices, and the inclusion of a heavily treated real-world population augment the external validity. The six-month observation period with structured interim visits permits assessment of the temporal kinetics of therapeutic response. The retrospective, single-arm observational design and absence of a comparator group preclude causal attribution of the observed improvements specifically to saroglitazar, particularly in the setting of background antidiabetic, statin, and SGLT2 inhibitor therapy. The universal use of statins, SGLT2 inhibitors, and multiple antidiabetic agents precludes isolation of saroglitazar's independent contribution to each outcome domain. Medication dose adjustments and intensity modifications of concomitant antidiabetic, lipid-lowering, and other disease-specific therapies during follow-up were not uniformly captured in the retrospective records, which may have contributed to residual confounding. Statin therapy was recorded as present in all patients at baseline; however, the specific statin molecule, dose, and intensity classification were not consistently available in the retrospective records, so stratification by low-, moderate-, or high-intensity statin therapy was not feasible. Subgroup analyses according to SGLT2 inhibitor, pioglitazone, and insulin use were not prespecified. Meaningful subgroup comparison was also limited by the distribution of background therapies, particularly the near-universal use of SGLT2 inhibitors. Future prospective studies should evaluate saroglitazar outcomes after stratification by major background therapies, including SGLT2 inhibitors, pioglitazone, and insulin. CAP score was analyzed as a continuous variable, and CAP-based steatosis categories, including normal, mild, moderate, or severe steatosis, were not available in the extracted retrospective dataset. Future analyses should report CAP-based steatosis categories in addition to the mean CAP score change. Generalizability may be limited by the potentially urban, healthcare-seeking study population. Long-term data beyond six months are unavailable, and hard cardiovascular or hepatic endpoints (e.g., liver biopsy, major adverse cardiovascular events) were not assessed.

## Conclusions

In this large retrospective real-world cohort of 600 Indian patients with T2DM and dyslipidemia, saroglitazar 4 mg demonstrated comprehensive, statistically significant, and clinically meaningful improvements in glycemic control, atherogenic dyslipidemia, hepatic enzyme levels, non-invasive markers of hepatic steatosis and fibrosis, renal function, and body weight over six months of treatment. The magnitude of triglyceride reduction and HDL-C increment, combined with an HbA1c reduction of 1.30 percentage points, positions saroglitazar as an agent associated with favorable real-world outcomes for the simultaneous management of the glycemic-lipid-hepatic triad in T2DM. These findings substantiate the real-world utility of saroglitazar 4 mg as an important add-on therapeutic option in the management of Indian patients with T2DM and mixed metabolic disease. Further randomized controlled trials with hard cardiovascular endpoints and long-term follow-up are warranted to establish the full therapeutic potential of this agent.
